# Incidence of lower respiratory tract infections in patients treated with post-cardiac arrest mild therapeutic hypothermia and selective digestive tract decontamination

**DOI:** 10.1186/cc9722

**Published:** 2011-03-11

**Authors:** NA Vellinga, EC Boerma, MA Kuiper

**Affiliations:** 1 Medisch Centrum Leeuwarden, the Netherlands

## Introduction

Mild therapeutic hypothermia (MTH) is known to have a neuroprotective effect after cardiac arrest (CA). Among the well-recognized side effects is an increased incidence of infections. A useful strategy in preventing lower respiratory tract infections (LRIs) during MTH is selective digestive tract decontamination (SDD). To this purpose, we examined the use of antibiotics and microbial flora in sputum in post-CA patients treated with MTH and SDD and compared this with the infection rate during MTH that has been reported in literature.

## Methods

We examined sputum (endotracheal aspirate) of all post-CA patients who were treated with MTH (32 to 34°C) during 24 hours after ICU admission and SDD/cefotaxim (SDD/CFT) in our 16-bed mixed ICU in a teaching hospital in the Netherlands in the period January 2007 to December 2008 (*n *= 55; male = 44, female = 11). Sputum was collected at ICU admission and several days later as part of our SDD/CFT routine. Between 24 and 48 hours after admission, body temperature was actively held below 37°C. LRI was defined as the presence of a potentially pathogenic microorganism (PPM) and the use of antibiotics other than SDD/CFT. The presence of *Candida albicans*/Candida spp. was considered colonisation and was treated with aerosol antifungal medication.

## Results

The in-hospital mortality in our cohort was 30.9%. As can be concluded from our results, in 59.5% of cases a PPM was present in the first sputum during SDD/CFT treatment after admission, with *C. albicans *being the most prevalent (23.6%). As compared with the sputum on admission, the cultures of the first sputum with SDD/CFT more often showed a monomicrobial isolate (25.5 vs. 40.5%). In sputum of 9/37 (24%) of our patients, a PPM (other than *C. albicans*/C. spp.) that justifies the use of antibiotics was present, with *S. aureus *being the most prevalent PPM (13.5%); 5/9 patients were treated with antibiotics, 1/9 received no additional antibiotics, 3/9 were lost to follow-up. Our results point towards a lower incidence of LRI in SDD/CFT-treated patients as compared with non-SDD/CFT-treated patients (88%) who were treated with MTH post-CA [1]. The incidence of LRI in our small cohort (24%) was also considerably lower as compared with a recent study by Nielsen and colleagues (48%) [2].

## Conclusions

Our results might point towards a beneficial role of SDD/CFT in preventing LRI during treatment with MTH.

## References

[B1] Nieuwendijk R Intensive Care Med 2008 34 S211 10.1007/s00134-007-0810-0

[B2] Nielsen N Crit Care Med 2010 in press

